# Are twindemics occurring?

**DOI:** 10.1111/irv.13090

**Published:** 2022-12-25

**Authors:** Emi Takashita, Shinji Watanabe, Hideki Hasegawa, Yoshihiro Kawaoka

**Affiliations:** ^1^ Research Center for Influenza and Respiratory Viruses National Institute of Infectious Diseases Tokyo Japan; ^2^ Division of Virology, Institute of Medical Science University of Tokyo Tokyo Japan; ^3^ Research Center for Global Viral Diseases National Center for Global Health and Medicine Research Institute Tokyo Japan; ^4^ Influenza Research Institute, Department of Pathobiological Sciences, School of Veterinary Medicine University of Wisconsin‐Madison Madison Wisconsin USA

**Keywords:** COVID‐19, influenza, SARS‐CoV‐2, viral interference

## Abstract

The emergence and spread of severe acute respiratory syndrome coronavirus 2 (SARS‐CoV‐2), which causes coronavirus disease (COVID‐19), prompted worldwide COVID‐19 surveillance. To investigate the impact of COVID‐19 on influenza activity, we used global surveillance data collected since 2019 to compare the number of cases positive for COVID‐19 and for influenza across 22 representative countries (Australia, Brazil, Canada, China, Egypt, France, Germany, India, Israel, Italy, Japan, Mexico, The Netherlands, The Philippines, Poland, The Republic of Korea, South Africa, Spain, Thailand, The United Kingdom, The United States, and Vietnam). Our results demonstrate alternating prevalence of SARS‐CoV‐2 and influenza virus.

The threat of concurrent COVID‐19 and influenza outbreaks is of great concern. Since 1952, the World Health Organization (WHO) Global Influenza Surveillance and Response System (GISRS) has conducted global influenza surveillance. The emergence and spread of severe acute respiratory syndrome coronavirus 2 (SARS‐CoV‐2), which causes coronavirus disease (COVID‐19), then prompted global COVID‐19 surveillance. The WHO GISRS has maintained its global influenza surveillance while using the sentinel network for the detection of SARS‐CoV‐2, and the available data indicate that the overall numbers of tests are not lower than those of previous years.^1^ To investigate the impact of COVID‐19 on influenza activity and vice versa, we compared the number of cases positive for COVID‐19 and for influenza from Week 1, 2019, through Week 45, 2022, in 22 countries representative of the WHO regions of Africa (South Africa), Eastern Mediterranean (Egypt), Europe (France, Germany, Israel, Italy, the Netherlands, Poland, Spain, and the United Kingdom), the Americas (Brazil, Canada, Mexico, and the United States), South‐East Asia (India and Thailand), and the Western Pacific (Australia, China, Japan, the Philippines, the Republic of Korea, and Vietnam) by using global surveillance data in the FluCov Dashboard (Nivel, https://www.nivel.nl/en/dossier-epidemiology-respiratory-viruses/flucov-dashboard) (Figure 1). The FluCov Dashboard has collected data to track SARS‐CoV‐2 and influenza virus activity in these countries from two sources: Our World in Data (https://ourworldindata.org) for SARS‐CoV‐2, and WHO FluNet (https://www.who.int/tools/flunet) for influenza virus since 2019.

**FIGURE 1:**
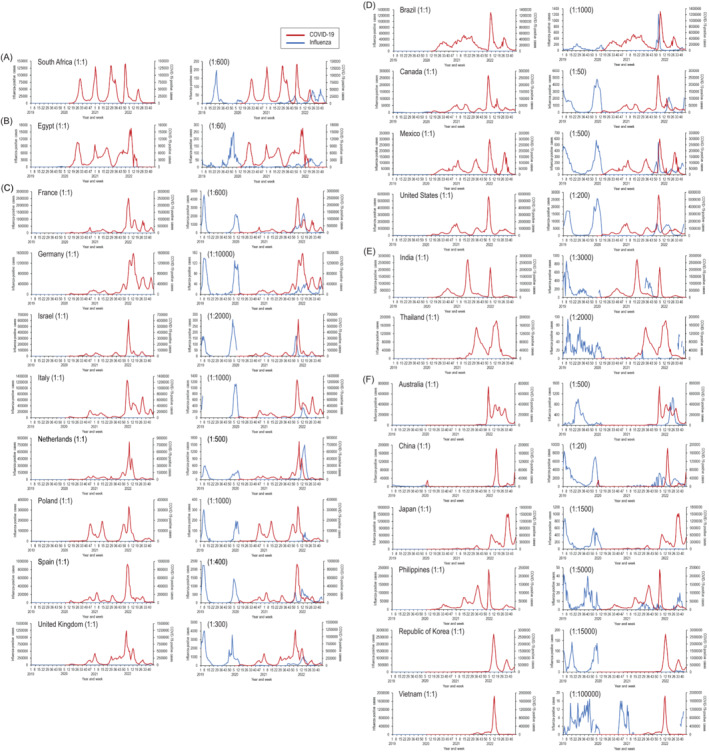
Number of cases positive for COVID‐19 and for influenza in 22 geographical representative countries from Week 1, 2019, through Week 45, 2022, according to the FluCov Dashboard (https://www.nivel.nl/en/dossier‐epidemiology‐respiratory‐viruses/flucov‐dashboard). The World Health Organization (WHO) regions of (A) Africa, (B) Eastern Mediterranean, (C) Europe, (D) Americas, (E) South‐East Asia, and (F) Western Pacific. Parentheses indicate the ratio of the left to right Y‐axis.

It should be noted that the number of influenza‐positive cases is substantially lower than the number of COVID‐19‐positive cases in all countries (Figure 1, left panels). Therefore, to facilitate our investigation of whether COVID‐19 outbreaks have any effect on influenza activity and vice versa, we adjusted the Y‐axis of positive cases for COVID‐19 and for influenza. Major reductions in influenza activity were observed after the emergence and spread of SARS‐CoV‐2, as has been reported elsewhere^1^ (Figure 1, right panels). In Japan and the Republic of Korea, low influenza activity has continued throughout the COVID‐19 pandemic. An apparent inverse correlation between COVID‐19 and influenza activities was observed in all other countries, except France, Germany, Italy, and the United Kingdom. To analyze the COVID‐19 and influenza activities from Week 1 to Week 45, 2022, in more detail, we compared them by adjusting their ratio to 1:500 (Figure 2A). Because the number of influenza‐positive cases was considerably low in Germany, we did not examine this country's data further. We found that the peak of influenza activity occurred in Week 13 in France, Week 12 in Italy, and Week 15 in the United Kingdom, respectively (Figure 2A, arrows). However, in general, the incidence of SARS‐CoV‐2 and influenza viruses increased in different regions of these countries each week (Figure 2B): Corsica (Santé Publique France, https://www.santepubliquefrance.fr/en/covid-19-epidemiological-update.-weekly-report.-week-13.-7-april-2022) and nine of 13 regions (Santé Publique France, https://www.santepubliquefrance.fr/maladies‐et‐traumatismes/maladies‐et‐infections‐respiratoires/grippe/documents/bulletin‐national/bulletin‐epidemiologique‐grippe‐semaine‐13.‐saison‐2021‐2022) in France; Lazio (Istituto Superiore di Sanità, https://www.salute.gov.it/imgs/C_17_monitoraggi_108_0_fileNazionale.pdf) and regions other than Lazio (Istituto Superiore di Sanità, https://www.salute.gov.it/portale/temi/documenti/virologica/Influnet_Virol_2022‐12.pdf) in Italy; and the South East (UK Health Security Agency, https://coronavirus.data.gov.uk/details/cases?areaType=nation&areaName=England) and the West Midlands (UK Health Security Agency, https://assets.publishing.service.gov.uk/government/uploads/system/uploads/attachment_data/file/1070098/Weekly_COVID‐19_and_Influenza_Surveillance_Graphs_w16.pdf) in the United Kingdom, respectively. In Week 13 in France, where the incidence rates for COVID‐19 and influenza activity in each region are available, the incidence rate of COVID‐19 was highest in Corsica (1979 cases per 100 000 population); influenza activity increased in nine of 13 regions, the exceptions being Corsica, Auvergne‐Rhône‐Alpes, Bourgogne‐Franche‐Comté, and Pays de La Loire. In Week 12 in Italy, where the number of COVID‐19‐positive cases and influenza activity in each region are available, the number of COVID‐19‐positive cases was highest in Lazio (57 780 cases); influenza‐positive cases were reported in 13 regions, but no positive cases were reported in Lazio. In Week 15 in the United Kingdom, where the number of cases positive for COVID‐19 and for influenza in each region are available, the number of COVID‐19‐positive cases was highest in the South East (27 366 cases); the highest number of influenza‐positive cases was reported in the West Midlands, whereas the number of influenza‐positive cases in the South East decreased from the previous week. These results indicate that SARS‐CoV‐2 and influenza virus may not reach their peaks at the same time in the same region.

**FIGURE 2:**
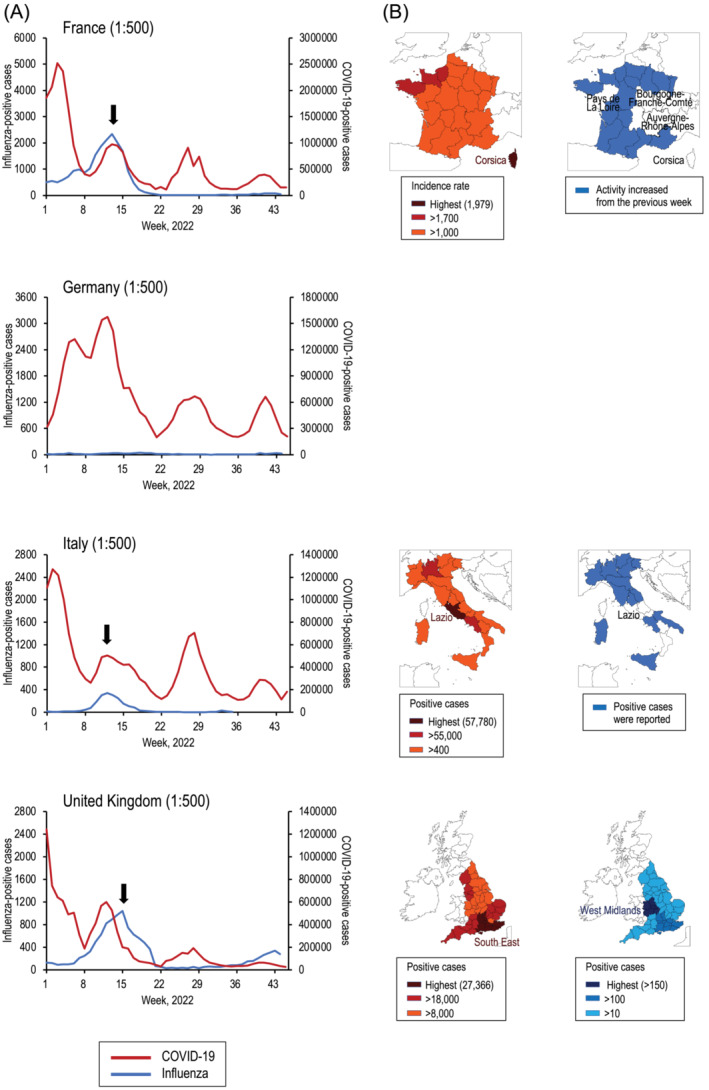
(A) Number of cases positive for COVID‐19 and for influenza in France, Germany, Italy, and the United Kingdom from Week 1 to Week 45, 2022, according to the FluCov Dashboard (https://www.nivel.nl/en/dossier‐epidemiology‐respiratory‐viruses/flucov‐dashboard). Parentheses indicate the ratio of the left to right Y‐axis. Arrows indicate the peak of influenza activity. (B) COVID‐19 (left panel) and influenza (right panel) activity in each geographical region at the influenza activity peak in each country. The left panels show the highest (1979 in France, 57 780 in Italy, and 27 366 in the United Kingdom) and next highest (>1700 in France, >55 000 in Italy, and >18 000 in the United Kingdom) regions for reported COVID‐19‐positive cases. The right panels show the influenza activity (France and Italy) or the highest (>150) and next highest (>100) regions for reported influenza‐positive cases (the United Kingdom).

Viral interference among influenza virus, rhinovirus, and other respiratory viruses has been reported at the host and population levels.^2^, ^3^, ^4^ A previous study showed that influenza infection reduced the risk of SARS‐CoV‐2 infection by 58%, suggesting possible interference between the two viruses.^5^ In vitro and in vivo viral interference between SARS‐CoV‐2 and influenza A(H1N1)pdm09, A(H3N2), or B/Victoria‐lineage virus has been reported.^6^, ^7^, ^8^, ^9^, ^10^, ^11^, ^12^ Simultaneous or sequential co‐infections with an early SARS‐CoV‐2 strain and influenza virus were analyzed by using reconstituted human airway epithelium^6^, ^7^, ^8^ and animal models such as Syrian hamsters^9^, ^10^, ^11^ and transgenic mice expressing human ACE2, the receptor for SARS‐CoV‐2.^12^ Although results may have depended on the influenza virus subtype and experimental conditions used, these studies suggested that SARS‐CoV‐2 replication is impaired by a prior infection with influenza A(H1N1)pdm09^7^, ^8^, ^9^, ^11^, ^12^ or influenza B virus,^7^ whereas A(H3N2) virus replication was impaired by SARS‐CoV‐2 infection.^10^ At the host level, viral interference between SARS‐CoV‐2 and influenza virus may be mediated by host innate immune responses, such as those activated by interferons and interferon‐stimulated genes.

SARS‐CoV‐2 continues to evolve by accumulating amino acid substitutions in its proteins. The WHO has defined B.1.1.7 (Alpha), B.1.351 (Beta), P.1 (Gamma), B.1.617.2 (Delta), and B.1.1.529 (Omicron) variants as Variants of Concern (VOCs) (https://www.who.int/activities/tracking-SARS-CoV-2-variants). The Omicron variant has been predominant worldwide since January 2022 (https://nextstrain.org/ncov/gisaid/global/all-time) and has further divided into subvariants, including VOCs BA.1, BA.2, BA.3, BA.4, and BA.5. However, whether viral interference occurs between Omicron variants and influenza virus remains unknown. Further analysis is needed to assess viral interference between currently circulating SARS‐CoV‐2 and influenza virus at the host and population levels.

## CONFLICTS OF INTEREST

None declared.

## AUTHOR CONTRIBUTIONS

**Emi Takashita:** Investigation; methodology; writing‐original draft. **Shinji Watanabe:** Writing‐review and editing. **Hideki Hasegawa:** Writing‐review and editing. **Yoshihiro Kawaoka:** Conceptualization; methodology; writing‐review and editing.

### PEER REVIEW

The peer review history for this article is available at https://publons.com/publon/10.1111/irv.13090.

## Data Availability

The data that support the findings of this study are openly available in FluCov Dashboard at https://www.nivel.nl/en/dossier-epidemiology-respiratory-viruses/flucov-dashboard.
